# Definitions of massive transfusion in adults with critical bleeding: a systematic review

**DOI:** 10.1186/s13054-023-04537-z

**Published:** 2023-07-05

**Authors:** Victor S. Lin, Emily Sun, Serine Yau, Chathuri Abeyakoon, Georgia Seamer, Simran Bhopal, Harriet Tucker, Carolyn Doree, Susan J. Brunskill, Zoe K. McQuilten, Simon J. Stanworth, Erica M. Wood, Laura Green

**Affiliations:** 1 Transfusion Research Unit, School of Public Health and Preventive Medicine, Monash University, Melbourne, Australia; 2 Faculty of Medicine, Dentistry and Health Sciences, University of Melbourne, Parkville, Australia; 3 Faculty of Medicine, Nursing, and Health Sciences, Monash University, Clayton, Australia; 4 Department of Clinical Haematology, Monash Health, Clayton, Australia; 5 Blizard Institute, Queen Mary University of London, London, UK; 6 Systematic Review Initiative, NHS Blood and Transplant, Oxford, UK; 7 Radcliffe Department of Medicine, University of Oxford, Oxford, UK; 8 National Institute for Health Research Biomedical Research Centre Haematology Theme, Oxford, UK; 9 Department of Haematology, Oxford University Hospitals NHS Foundation Trust, Oxford, UK; 10 NHS Blood and Transplant, London, UK; 11 Barts Health NHS Trust, London, UK

**Keywords:** Massive transfusion, Bleeding, Haemorrhage, Systematic review, Randomised controlled trials, Definition

## Abstract

**Background:**

Definitions for massive transfusion (MT) vary widely between studies, contributing to challenges in interpretation of research findings and practice evaluation. In this first systematic review, we aimed to identify all MT definitions used in randomised controlled trials (RCTs) to date to inform the development of consensus definitions for MT.

**Methods:**

We systematically searched the following databases for RCTs from inception until 11 August 2022: MEDLINE, Embase, Cochrane Central Register of Controlled Trials (CENTRAL), PubMed, Cumulative Index to Nursing and Allied Health Literature, and Transfusion Evidence Library. Ongoing trials were sought from CENTRAL, ClinicalTrials.gov, and World Health Organisation International Clinical Trials Registry Platform. To be eligible for inclusion, studies had to fulfil all the following three criteria: (1) be an RCT; (2) include an adult patient population with major bleeding who had received, or were anticipated to receive, an MT in any clinical setting; and (3) specify a definition for MT as an inclusion criterion or outcome measure.

**Results:**

Of the 8,458 distinct references identified, 30 trials were included for analysis (19 published, 11 ongoing). Trauma was the most common clinical setting in published trials, while for ongoing trials, it was obstetrics. A total of 15 different definitions of MT were identified across published and ongoing trials, varying greatly in cut-offs for volume transfused and time period. Almost all definitions specified the number of red blood cells (RBCs) within a set time period, with none including plasma, platelets or other haemostatic agents that are part of contemporary transfusion resuscitation. For completed trials, the most commonly used definition was transfusion of ≥ 10 RBC units in 24 h (9/19, all in trauma), while for ongoing trials it was 3–5 RBC units (*n* = 7), with the timing for transfusion being poorly defined, or in some trials not provided at all (*n* = 5).

**Conclusions:**

Transfusion of ≥ 10 RBC units within 24 h was the most commonly used definition in published RCTs, while lower RBC volumes are being used in ongoing RCTs. Any consensus definitions should reflect the need to incorporate different blood components/products for MT and agree on whether a ‘one-size-fits-all’ approach should be used across different clinical settings.

**Supplementary Information:**

The online version contains supplementary material available at 10.1186/s13054-023-04537-z.

## Introduction

Major bleeding requiring blood transfusion can occur in almost any clinical setting and is a medical emergency [1]. The impact of blood component transfusion and other haemostatic interventions on improving outcomes of bleeding patients has been a topic of increasing research interest, as demonstrated by multiple recent randomised controlled trials (RCT) [2, 3], giving momentum to trials evaluating haemostatic resuscitation. Several studies have also shown that morbidity and mortality rates increase as transfusion requirements increase [4–6],﻿ emphasising the importance of blood transfusion requirement as a predictive measure of patient outcome. Many recent RCTs are now using transfusion requirement as a primary or key secondary outcome to evaluate interventions (e.g. iTACTIC [7]), recognising in part the convenience of quantification of blood component needs as well as the relevance of this outcome to patients and healthcare resources. However, there is no standardised or universally accepted definition for massive transfusion (MT) in major bleeding, which makes comparing or synthesising the results of such trials difficult.

Historically, MT has often been arbitrarily defined as transfusion of ≥ 10 units of whole blood or red blood cells (RBC) within 24 h, as an approximation of the replacement of ≥ 1 total blood volume [8, 9]. However, this definition has several limitations: it fails to capture patients who die early, before the 24-h or 10-unit threshold is reached, resulting in survivorship bias [8] and it is a retrospective definition. In the last decade, other more dynamic definitions for MT have been introduced, including transfusion of ≥ 5 RBC units in 4 h, the replacement of ≥ 50% of total blood volume in 3 h, or ≥ 3 RBC units during any 1-h period in the first 24 h after hospital arrival, which capture the acuity of the transfusion response [10–12]. However, none of these definitions take into account other blood components/products, such as plasma and platelets, or other haemostatic agents that are key parts of contemporary transfusion resuscitation [13]. As the number of studies on bleeding increases, the absence of an agreed-upon international consensus for definitions of MT adds even more complexities to designing research studies, synthesising findings, and comparing research outcomes and clinical services across countries.

In collaboration with the International Society for Blood Transfusion Clinical Working Party, and the International Collaboration for Transfusion Medicine Guidelines, we performed a systematic review of the literature to scope out the most common MT definitions used in RCTs of adult patients with critical bleeding to inform broader discussions around the need for consensus definitions of MT.

## Methods

This scoping review was conducted in accordance with published guidelines for conducting scoping reviews [14].

### Search strategy

To identify definitions for MT used in RCTs published to date, an information specialist (CD) performed a comprehensive search using MeSH index and free-text terms of the following databases: MEDLINE, Embase, Cochrane Central Register of Controlled Trials (CENTRAL) in the Cochrane Library, PubMed, Cumulative Index to Nursing and Allied Health Literature (CINAHL), and Transfusion Evidence Library. Ongoing trials were sought from CENTRAL, ClinicalTrials.gov, and World Health Organisation (WHO) International Clinical Trials Registry Platform (ICTRP). The search was performed from inception until 11 August 2022. No language restriction was placed on the search, and any papers not published in English were translated. The full search strategy is available in Additional file 1.

### Selection criteria

To be eligible for inclusion, studies were required to meet all the following three criteria: (1) be an RCT; (2) include an adult patient population with major bleeding who had received, or were anticipated to receive, MT in any clinical setting; and (3) specify a definition for MT as an inclusion criterion or outcome measure. Conference abstracts, opinion pieces, clinical guidelines, narrative reviews, post hoc analyses, and studies that included only paediatric or non-human subjects were excluded. In cases where trial protocol and trial results were published separately, the publication with the trial results was included. The reason for including only randomised controlled trials was twofold: (1) to include only high-quality studies, and (2) to make the process of review manageable for the resources available. We acknowledge that many other studies have been performed using a wide range of definitions, but many of these were likely not pre-planned or validated.

The search criteria used is provided in Appendix 1 under Additional file 1. All citations identified by the search strategy were uploaded to Covidence, a web-A﻿ll citations identified by the search strategy were uploaded to Covidence, a web-based software platform, and the titles and abstracts were screened for eligibility by two independent reviewers (VL, HT, CA, ES, GS, SY, SB, or CD). The same reviewers then independently screened the full texts for inclusion. Any disagreements were resolved through discussion or referred to a third reviewer (LG). The reference lists of any relevant studies were also reviewed to identify any additional RCTs.

### Data extraction and analysis

Data were independently extracted onto standardised and pre-piloted forms by pairs of two reviewers (VL and ES, CA and SB, or GS and SY). The following data were extracted: (1) definition of MT used; (2) types of blood product transfused; (3) relevant clinical specialty; (4) patient demographics; (5) interventions being compared; (6) sample size of study. Any disagreements were resolved through discussion or referred to a third reviewer (LG).

## Results

### Study selection

**Fig. 1:**
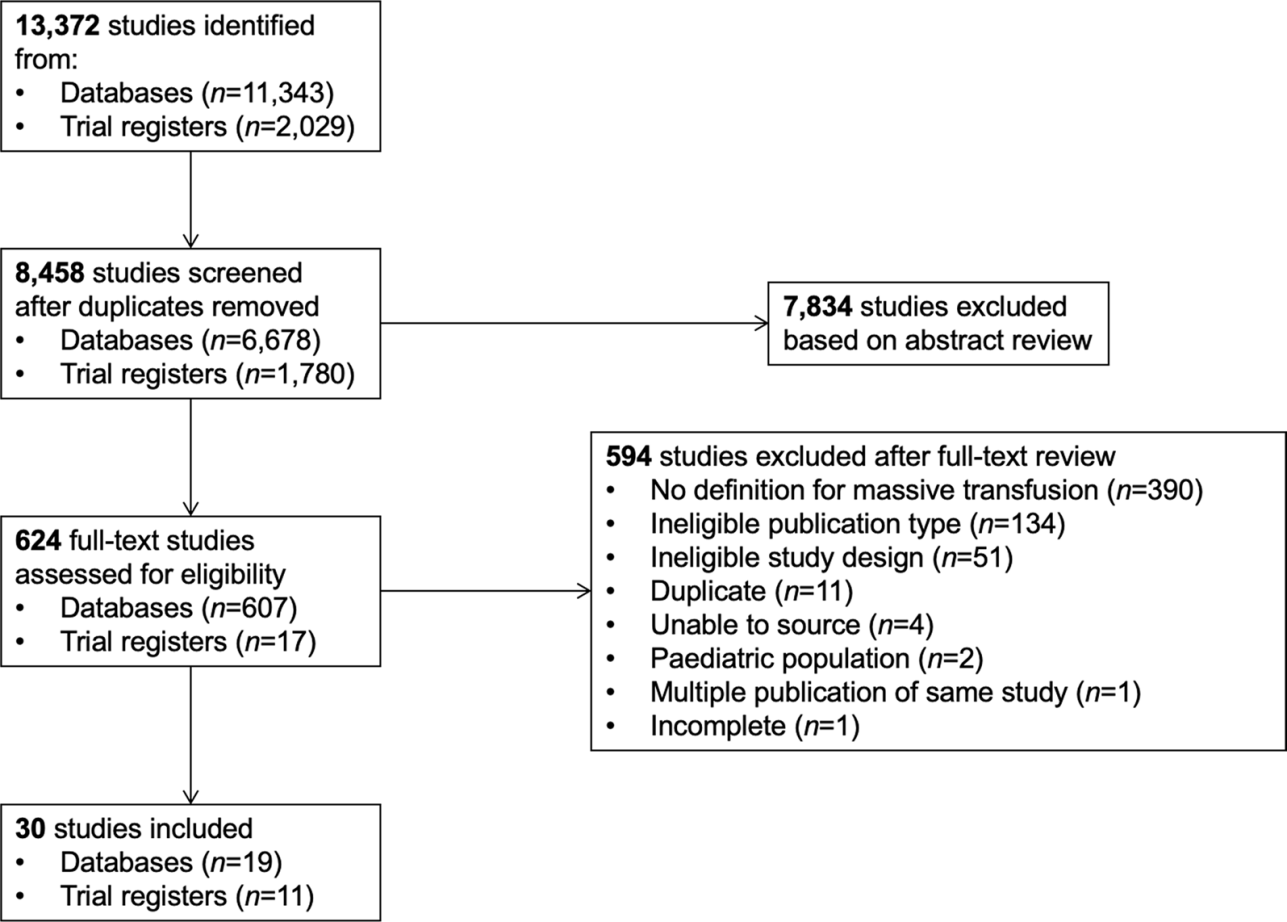
Selection of studies in the scoping review

**Fig. 2:**
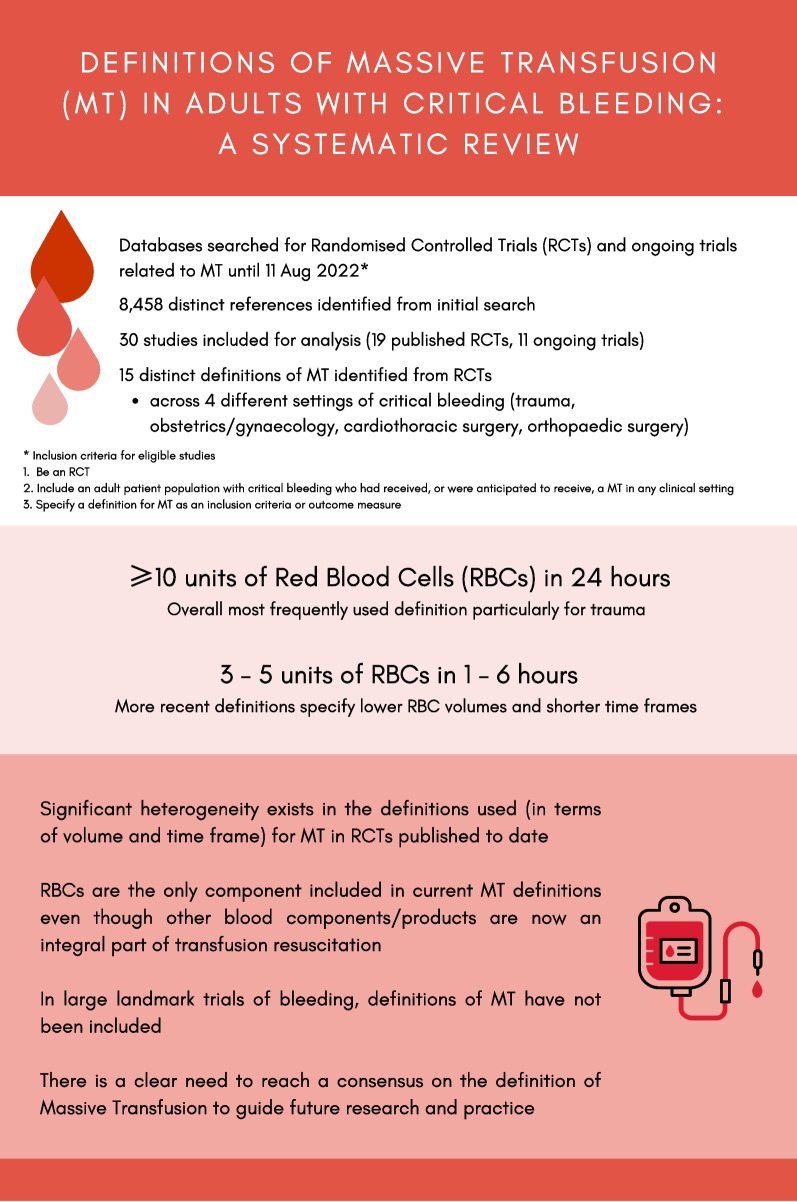
Summary of key findings from our scoping review

The PRISMA flow diagram of included and excluded studies is shown in Fig. 1. Our search strategy identified 13,372 references (11,343 published studies and 2,029 ongoing trials). Once duplicates were removed, a total of 8,458 references were screened for eligibility based on title and abstract, which identified 624 references that underwent full-text assessment. Of these, 594 references were excluded, with the main reasons for exclusion being lack of a definition for MT (*n* = 390), ineligible publication type (*n* = 134), and ineligible study design (*n* = 51). The remaining 30 studies were included in the review. Of the 30 studies, 19 were published RCTs [2, 7, 15–31], including three published trial protocols [28–30], and 11 were ongoing RCTs. These will be described separately in the text and in Tables 1 and 2. A summary of all MT definitions used in RCTs to date is included in Table 3. A summary of all key findings from our study is presented in Fig. 2.

**Table 1:** Summary of published randomised controlled trials included in the review

Study	Clinical setting	Country	Intervention	Control	Total number of subjects recruited	MT definition
Bulger 2008	Trauma	USA	Hypertonic saline/dextran	Lactated Ringer solution	209	≥ 10 U RBC/24 h
Hauser 2010 (CONTROL)	Trauma	Multinational	rFVIIa	Placebo	573	≥ 10 U RBC/24 h
Nascimento 2013 (TRFL)	Trauma	Canada	Fixed-ratio transfusion	Laboratory-guided transfusion	78	≥ 10 U RBC/24 h
Holcomb 2015 (PROPPR)	Trauma	Multinational	1:1:1 RBC:FFP:PLT	1:1:2 RBC:FFP:PLT	680	≥ 10 U RBC/24 h
Innerhofer 2017 (RETIC)	Trauma	Austria	FFP	CFC	100	≥ 10 U RBC/24 h
Sperry 2018 (PAMPer)	Trauma	USA	Plasma	Standard care	501	≥ 10 U RBC/24 h
Baksaas-Aasen 2021(iTACTIC)	Trauma	Multinational	VHA	CCT	396	≥ 10 U RBC/24 h
Bouzat 2021 (PROCOAG)	Trauma	France	Four-factor PCC	Saline	N/A	≥ 3 U RBC/1 h or ≥ 10 U RBC/24 h
Jost 2022 (PREHO-PLYO)	Trauma	France	Plasma	Control	134	≥ 10 U RBC/24 h
Moore 2018 (COMBAT)	Trauma	USA	Plasma	Saline	125	> 10 U RBC
Boffard 2005	Trauma	Multinational	rFVIIa	Placebo	277	> 20 U RBC
Nathens 2006	Trauma	USA	Leukoreduced transfusion	Non-leukoreduced	268	> 6 U/24 h
Martinaud 2019 (T-STORHM)	Trauma	France	Whole blood	Component therapy	N/A	≥ 4 U RBC/6 h
Girdauskas 2010	Cardiothoracic surgery	Germany	Treatment group	Control group	56	> 20 U blood
Vlasov 2020 (ALBICS)	Cardiothoracic surgery	Finland	4% albumin	Ringer's acetate solution	N/A	≥ 5 U RBC
Klein 2001	Cardiothoracic surgery	Multinational	Roller pump	BioPump	1000	> 5 U blood
Locker 1997	Orthopedic surgery	Austria	PGE1	Placebo	45	> 10 U RBC
Ngichabes 2015	Obstetrics /gynaecology	Kenya	TXA	Ornipressin	34	> 5 U blood
Reed 1986	Mixed	USA	PLT	FFP	33	≥ 12 U blood/12 h

*MT* massive transfusion, *USA* United States of America, *U* units, *RBC* red blood cells, *h* hours, *rFVIIa* recombinant factor VIIa, *FFP* fresh frozen plasma, *PLT* platelets, *CFC* coagulation factor concentrates, *VHA* viscoelastic haemostatic assays, *CCT* conventional coagulation tests, *PCC* prothrombin complex concentrate, *PGE1* prostaglandin E1, *N/A* not applicable

**Table 2:** Summary of ongoing randomised controlled trials included in the review

Study title (clinical trial number)	Date first posted	Country	Clinical setting	Intervention	Control	Total number of subjects	MT definition
The efficacy and safety of preoperative misoprostol in blood-loss reduction during myomectomy (NCT03509168)	26/4/18	Uganda	Gynaecology	Misoprostol	No Misoprostol	46	> 5 U blood
Glove-loaded Foley's catheter tamponade for Cesarean section for placenta previa (NCT03570723)	27/6/18	Egypt	Obstetrics	Stepwise uterine devascularisation	A glove-loaded Foley's catheter	120	> 3 U RBC
Reducing blood loss during Cesarean hysterectomy for placenta accreta spectrum (NCT03570710)	27/6/18	Egypt	Obstetrics	TXA	Placebo	80	> 4 U RBC
Patient blood management for massive obstetric hemorrhage (NCT03784794)	24/12/18	Mexico	Obstetrics	TEG-guided transfusion	Standard algorithm	100	≥ 10 U RBC/24 h
Anticipatory management vs standard management of postpartum haemorrhage (CN-01883655)	25/7/11	Sri Lanka	Obstetrics	Anticipatory management protocol	Standard management protocol	252	> 5 U blood
Clinical utility of thromboelastography in guiding prophylaxis of venous thromboembolism following trauma (NCT01050153)	15/1/10	USA	Trauma	TEG-guided thromboprophylaxis	Standard of care	50	> 10 U RBC/6 h
Freeze-dried plasma in the initial management of coagulopathy in trauma patients (NCT02750150)	25/4/16	France	Trauma	Freeze-dried plasma	FFP	42	≥ 4 U RBC/6 h
Dose section phase II study to compare the inhibition of blood loss in patients undergoing primary heart surgery and treatment with the investigational drug MDCO-2010, the active comparator tranexamic acid, or placebo (CN-01801695)	24/11/11	Multinational	Cardiothoracic surgery	Antifibrinolytic MDCO-2010	TXA and placebo	44	> 5 U RBC/24 h
Patient mobility and outcomes after cardiac surgery (NCT03806257)	16/1/19	USA	Cardiothoracic surgery	Enhanced physical therapy protocol	Control	220	> 10 U RBC
Intervention study about the additive infusion of concentrated fibrinogen preparation and the amount of blood transfusion for the massive hemorrhage case (UMIN000019027)	15/9/15	Japan	Trauma and gastrointestinal	Concentrated fibrinogen	No concentrated fibrinogen	70	≥ 10 U RBC
Evaluating of the Hospital Universitario de Canarias massive transfusion protocol (NCT03074890)	9/3/17	Spain	Mixed	1:1:1 ratio of RBC:FFP:PLT and conventional coagulation tests guiding resuscitation	No intervention	30	≥ 3 U RBC/1 h, 10 U RBC

*MT* massive transfusion, *U* units, *RBC* red blood cells, *TXA* tranexamic acid, *TEG* thromboelastogram, *h* hours, *USA* United States of America, *FFP* fresh frozen plasma, *PLT* platelets

**Table 3:** Summary of all definitions for massive transfusion used in randomised controlled trials to date

	MT definition	Total number of RCTs using the definition
1	≥ 10 U RBC/24 h	9
2	> 5 U blood	4
3	> 10 U RBC	3
4	≥ 4 U RBC/6 h	2
5	≥ 3 U RBC/1 h or ≥ 10 U RBC/24 h	2
6	> 20 U RBC	1
7	> 6 U/24 h	1
8	> 20 U blood	1
9	≥ 5 U RBC	1
10	≥ 12 U blood/12 h	1
11	> 3 U RBC	1
12	> 4 U RBC	1
13	> 10 U RBC/6 h	1
14	> 5 U RBC/24 h	1
15	≥ 10 U RBC	1
	**Total**	30

*MT* massive transfusion, *RCT* randomised controlled trial, *U* unit, *RBC* red blood cell, *h* hour

### Published RCTs

The year of publication for published RCTs spanned from 1986 to 2022. All published RCTs were written in English and were conducted in diverse geographical locations. Eight were conducted across multiple continents, with a further 11 in Europe, 10 in North America, four in Africa, two in Asia, and one in South America. The trials were conducted in diverse clinical settings, with the most common clinical setting being trauma (13/19), followed by cardiothoracic surgery (3/19) (Table 1). The total sample size for all published trials was 4509 patients, with the sample size for each trial ranging from 33 to 680 patients. The most common interventions studied was blood transfusion (8/19), particularly plasma, followed by haemostatic agents (3/19) and others. The most common definition for MT was transfusion of ≥ 10 units of RBC in 24 h (9/19 RCTs, all in trauma), followed by 5 units of RBC or blood (*n* = 3), and 10 units of RBC (*n* = 2). Time intervals for the latter two definitions were not provided. None of the MT definitions included other blood components (e.g. plasma or platelets) or blood products (e.g. fibrinogen concentrate, prothrombin complex concentrate) or haemostatic agents (e.g. recombinant factor VIIa).

### Ongoing RCTs

Of the 11 ongoing trials, three trials were conducted in Africa, two in Europe, two in Asia, two in USA, one in South America, and one multinational. The most common clinical setting was obstetrics (*n* = 4), followed by cardiothoracic surgery (*n* = 2) and trauma (*n* = 2) (Table 2). The total sample size for all ongoing trials was 1,054 patients, with the sample size for each trial ranging from 30 to 252 patients. The most common definition used for MT in the ongoing trials was 3–5 units of RBC (*n* = 7) and ≥ 10 units of RBC (*n* = 5); the timing for transfusion was poorly defined, with some trials not providing this at all (*n* = 6).

## Discussion

In this first-ever scoping review of MT definitions used in RCTs to date, we identified a total of 15 different definitions for MT, with trauma being well represented as the main clinical setting for published trials, while obstetrics was the most common clinical setting for ongoing trials. The number of ongoing trials providing definitions for MT also confirms the common interest of applying definitions of transfusion needs in trials of bleeding. All identified definitions used transfusion of RBCs or whole blood as part of the MT definition either within a set time, or with no specified time-frame. Transfusion of ≥ 10 units of RBC within 24 h was the most common definition used in published RCTs, particularly for trauma, whereas lower transfusion volumes were used (e.g. 5 units of RBC) in ongoing RCTs.

Treatment of major haemorrhage that results in large volumes of allogeneic blood transfusion is not only associated with significant morbidity and mortality for patients, but also consumes considerable healthcare resources. Therefore, ‘large volume transfusion’ (or MT) is an attractive outcome measure, which encompasses both patients’ wellbeing and resource utilisation. Indeed, in recent trials, MT has been used as a primary outcome measure for evaluating efficacy of different interventions. However, the lack of an agreed-upon definition for MT makes comparisons between trials and clinical services difficult.

We identified significant heterogeneity in the definitions of MT reported in published RCTs, and almost all definitions were based on the number of RBC units transfused within a set time interval, with cut-offs for volume and time parameters varying greatly across trials. The last two decades have seen significant changes in transfusion resuscitation of bleeding patients across different disciplines, driven mainly by studies in trauma, with plasma, platelets, and fibrinogen replacement therapies being given early and in high ratios to RBC. Yet in our review, for both completed and ongoing trials, we did not see these blood components or products being featured in definitions of MT. The recent international survey conducted by our group as part of the work to harmonise definitions for MT also showed huge variation in MT definition used between countries and, in some instances, the definitions between major bleeding and MT were being blurred [32].﻿ Despite these variations, in this review and in the survey, transfusion of ≥ 10 units of RBC in 24 h emerged as the most used definition. While this definition has the advantage of being simple to adopt and apply, it does not reflect contemporary transfusion practices, which emphasise higher ratios of non-RBC components earlier in resuscitation.

In our review, we noted a move away from the more frequent definition of ≥ 10 units of RBC in 24 h for MT between published RCTs and ongoing RCTs, particularly for obstetrics, in which transfusion of ≥ 10 RBC units is a very rare event [33]. Even in trauma, where the definition of ≥ 10 RBC units in 24 h was utilised the most in the published RCTs, its incidence over the last decade has reduced significantly, due to the advent of early damage-control resuscitation (DCR) [34]. Therefore, in recent years, other definitions are now being increasingly used, reflecting current practices, the timing of interventions, and the timing of mortality from haemorrhage. For example, a study by Zatta and colleagues comparing three different MT definitions based on RBC only showed that transfusion of ≥ 5 units RBCs in 4 h is a more inclusive definition than ≥ 10 units of RBC in 24 h [10]. The ongoing RCT being conducted in the UK comparing whole blood versus blood component therapy in treating prehospital trauma bleeding patients (SWIFT) has defined MT as transfusion of ≥ 10 units of any blood components or products (e.g. RBC, plasma, platelets, cryoprecipitates) in the first 24 h of bleeding. A recent Delphi study proposed a new consensus definition of MT in adult trauma of ≥ 4 units of any blood component given within 2 h of injury [35]. A post hoc analysis of the PROPPR trial showed that other MT definitions like critical administration threshold of ≥ 3 units of RBCs in 1 h, and a resuscitation intensity of ≥ 4 units of blood product (taking into account RBC, plasma, crystalloid and colloid) are better at predicting early mortality than the definition of ≥ 10 units of RBCs in 24 h [36].

The implications of our review are the need for a more widely accepted definition of MT, as the transfusion of ‘ ≥ 10 units of RBC in 24 h’ does not reflect current transfusion practices and may no longer be fit for purpose. Any consensus definitions should reflect the need for modification by different clinical settings and may need to incorporate different types of blood components/products or fluids, since they are often administered as part of contemporary resuscitation and, while frequently not captured in existing definitions, constitute an important component of ‘transfusion’ [37]. Clearly, a major focus on consensus definitions should include a broader discussion about time points for the evaluation of MT in bleeding patients.

The UK audit of major bleeding in 2018 showed that surgery (28%), followed by obstetrics (21%), gastrointestinal bleeding (20%), and trauma (17%) were the most common causes of major haemorrhage [38], and this pattern has also been reported by other studies [10, 12, 39]. In this review, most of the published trials were in trauma, while for ongoing trials obstetrics was the dominant setting. We did not identify any RCTs in general surgery, vascular surgery, or gastrointestinal bleeding, despite these making up the majority of major bleeding cases in hospitals. Notably, several landmark trials on critically bleeding patients, such as the CRASH-2 [40], WOMAN [41], and HALT-IT [42] trials were excluded from our review, as they did not provide a specific definition of MT.

As we see an increasing number of RCTs being performed in major bleeding, we should be able to identify the rates of severe bleeding that require a large volume blood transfusion from these trials, so we can better understand the impact of different interventions on this important outcome. For example, the harmonisation of major bleeding definition by the International Society on Thrombosis and Haemostasis in surgical and non-surgical patients was crucial to evaluating and quantifying the safety of different direct antithrombotic agents in both surgical and medical settings. We hope that similar benefits will arise by harmonising definition for MT.

**Fig. 3:**
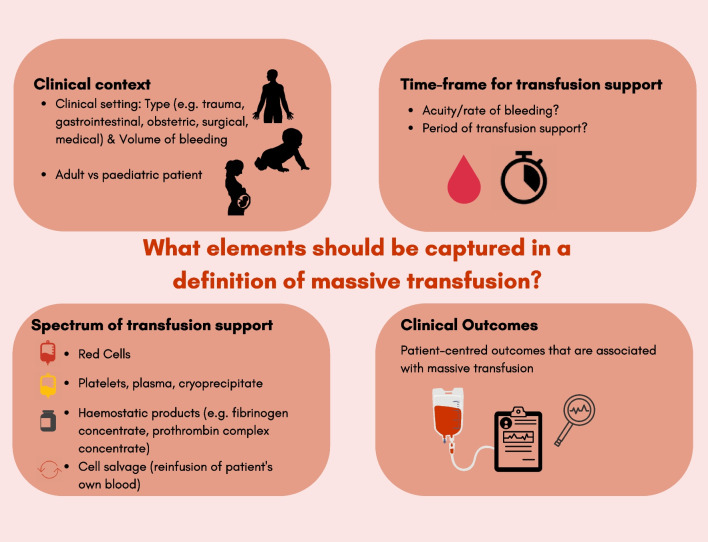
Key elements to be captured in a definition of massive transfusion

The next step should be to move to a stakeholder consultation and a Delphi process to reach a consensus on an MT definition in all relevant clinical settings, with the hope of reconciling the diverse range of definitions that have appeared in the transfusion literature to date. Based on our review, we propose that the following considerations be made for reaching a consensus for MT definitions: involvement of a wider range of stakeholders, including patients; consideration of the age of patients (e.g. adult or paediatric); consideration of a specific clinical setting (e.g. trauma, gastrointestinal, obstetric, surgical or medical); inclusion of other blood components (e.g. plasma, platelets); inclusion of other blood products (e.g. fibrinogen concentrate, prothrombin complex concentrate, cell salvage); and associating the timepoint for defining MT with patient-centred outcomes (e.g. mortality, organ failure, hospital stay) (Fig. 3).

### Strengths and limitations

To the best of our knowledge, this is the first scoping review examining MT definitions used in RCTs to date. The main strength of this review is the systemic approach taken, with no language restrictions placed on the search and two independent reviewers evaluating all references. The main limitation was the strictness of our inclusion criteria. Our review included only RCTs and excluded all other study designs, including cohort and case–control studies. The inclusion of all study designs, although informative for a scoping review, would have resulted in an unmanageably large number of studies to be reviewed for the resources that we had available. Our review focussed only on the adult population; a separate scoping review by our group will examine MT definitions in the paediatric population. Our review only examined studies in which the term ‘massive transfusion’ was featured and defined. Theoretically, this could have resulted in the exclusion of studies that used a different term to refer to a similar concept of ‘large volume transfusion’.

## Conclusion

In 19 completed RCTs and 11 ongoing RCTs, we identified 15 definitions of MT used as either an inclusion criterion or an outcome measure in adult patients with major bleeding. This heterogeneity in definitions makes it challenging to synthesise research outcomes as well as compare and optimise transfusion practices between centres and countries. Trauma and obstetrics were the most common settings where definitions for MT were reported, with no trials measuring MT as an outcome in surgical or gastrointestinal bleeding. The most commonly used definition for MT was transfusion of ≥ 10 units of RBC in 24 h for completed trials, while for ongoing trials, a lower RBC volume over a shorter time period is being used to define MT, particularly for obstetrics. Almost all MT definitions used RBC volume, while other blood components and products were not included in the MT definition, despite forming a key part of current transfusion practice for resuscitating bleeding patients. There is a clear need to harmonise definitions for MT to allow comparison of studies and services, optimisation of patient outcomes, and improved usage of healthcare resources.

## Supplementary Information

**Additional file** **1**. Appendix.

## Data Availability

Data supporting the results are presented in the article. The datasets used and/or analysed during the current study are available from the corresponding author on reasonable request.

## Abbreviations

MT: Massive transfusion
RCT: Randomised controlled trial
RBC: Red blood cells
DCR: Damage-control resuscitation
