# Supplementary material for: Definitions of massive transfusion in adults with critical bleeding: a systematic review
Source: Crit Care. 2023 Jul 5;27:265. doi: 10.1186/s13054-023-04537-z (PMC10324127; doi:10.1186/s13054-023-04537-z)
Supplement: Supplementary file 1 — Additional file 1. Appendix. [file 13054_2023_4537_MOESM1_ESM.docx]

**Supplementary Material**

**Appendix 1: Full search strategy**

**MEDLINE**

1. exp Blood Transfusion/

2. (transfus* or pretransfus* or posttransfus* or retransfus* or packed red cells or packed red blood cells or RBC* or PRBC* or packed red blood cells or platelets or fresh plasma or frozen plasma or FFP or platelet concentrate* or blood product* or blood component* or blood management or blood therapy or blood replacement).ti,kf.

3. (pretransfus* or posttransfus* or retransfus* or RBC transfusion* or pRBC transfusion* or red cell transfusion* or red blood cell transfusion* or packed cell transfusion* or blood transfusion* or platelet transfusion* or plasma transfusion* or fresh plasma or frozen plasma or FFP or platelet concentrate* or packed red cell* or packed red blood cell* or ((packed cell* or allogeneic) adj2 transfus*)).ab.

4. ((transfus* or whole blood or component therapy or blood component* or blood product* or blood replacement or allogeneic blood or allogenic blood or red cell* or red blood cell* or thawed plasma or liquid plasma or lyophili?ed plasma or platelets or packed cell* or packed red cell* or packed red blood cell* or RBC* or PRBC*) adj4 (use* or usage* or utili?ation or requir* or replac* or administ* or need* or protocol*)).tw,kf.

5. (((haemostatic or hemostatic) adj1 resuscitation) or haemotherap* or hemotherap*).tw,kf.

6. (total blood volume and transfus*).tw,kf.

7. ((red cell* or red blood cell* or RBC* or PRBC* or blood transfus* or transfus* ratio) adj5 (FFP or fresh plasma or frozen plasma or plasma transfusion*)).tw,kf.

8. or/1-7

9. Shock, Hemorrhagic/

10. exsanguinat*.mp.

11. Postpartum Hemorrhage/

12. ((major* or massive* or critical* or severe* or life-threatening or catastrophic* or uncontrolled or postpartum* or trauma*) adj3 (hemorrhag* or haemorrhag* or bleed* or bloodloss* or blood loss*)).tw,kf.

13. ((haemorrhagic or hemorrhagic or hypovolemic or hypovolaemic) adj shock).tw,kf.

14. or/9-13

15. exp Multiple Trauma/

16. exp Shock, Traumatic/

17. ((major* or life-threatening or multip* or severe* or serious* or catastrophic* or critical* or massive* or penetrating) adj3 (trauma* or posttrauma* or postrauma* or injury or injuries or injured or postinjur*)).tw,kf.

18. ((trauma* adj3 coagulopath*) or trauma* resuscitation or polytrauma*).tw,kf.

19. (severe* trauma* or major* trauma* or massive* trauma* or multipl* trauma* or polytrauma* or life-threatening trauma* or severe* injur* or serious* injur* or massive* injur* or critical* injur* or major* injur* or life-threatening injur* or multipl* injur*).ti,kf.

20. or/15-19

21. Aortic Rupture/

22. ((ruptur* or burst*) adj3 aort*).tw,kf.

23. or/21-22

24. 14 or 20 or 23

25. 8 and 24

26. ((massive* or mass or supermassive* or supramassive* or major) adj3 transfus*).tw,kf.

27. ((massive* dos* or massive* volume* or high dos* or high volume* or large dos* or large volume*) adj3 (red cell* or red blood cell* or RBC* or packed red cell* or packed red blood cell* or PRBC* or plasma or FFP or platelet* or transfus*)).tw,kf.

28. ((major bleeding or major haemorrhag* or major hemorrhag* or massive* bleeding or massive* haemorrhag* or massive* hemorrhag*) adj3 (management* or protocol*)).tw,kf.

29. ((trauma* or neurotrauma* or polytrauma* or posttrauma* or substantial* injur* or penetrating injur* or severe* injur* or serious injur* or massive* injur* or critical* injur* or major* injur* or life-threatening injur* or multiple injur*) adj6 (transfus* or whole blood or component therapy or blood component* or blood product* or blood replacement or allogeneic blood or allogenic blood or red cell* or red blood cell* or FFP or fresh plasma or frozen plasma or thawed plasma or liquid plasma or platelets or platelet concentrate* or packed cells or RBC* or PRBC*)).tw,kf.

30. or/25-29

31. Meta-Analysis/ or Network Meta-Analysis/

32. Systematic Review.pt.

33. "Systematic Reviews as Topic"/ or "Meta-Analysis as Topic"/

34. ((meta analy* or metaanaly*) and (trials or studies)).ab.

35. (meta analy* or metaanaly* or evidence-based).ti.

36. ((systematic* or evidence-based) adj2 (review* or overview*)).tw,kf.

37. (evidence synthes* or cochrane or medline or pubmed or embase or cinahl or cinhal or lilacs or "web of science" or science citation index or scopus or search terms or literature search or electronic search* or comprehensive search* or systematic search* or published articles or search strateg* or reference list* or bibliograph* or handsearch* or hand search* or manual* search*).ab.

38. Cochrane Database of systematic reviews.jn.

39. ((additional adj (papers or articles or sources)) or (relevant adj (journals or articles))).ab.

40. ((electronic* or online) adj (sources or resources or databases)).ab.

41. network meta-analys*.tw,kf.

42. or/31-41

43. Review.pt.

44. Randomized Controlled Trials as Topic/

45. selection criteria.ab. or critical appraisal.ti.

46. (data adj (abstraction or extraction or analys*)).ab.

47. exp Randomized Controlled Trial/

48. or/44-47

49. 43 and 48

50. 42 or 49

51. (Controlled Clinical Trial or Clinical Trial Protocol).pt.

52. exp Randomized Controlled Trial/

53. (randomi* or randomly or placebo).tw,kf.

54. trial.ti,kf.

55. Clinical Trials as Topic/

56. Clinical Trial, Phase III/ or ("phase 3" or "phase3" or "phase III" or P3 or "PIII").tw,kf.

57. or/51-56

58. 50 or 57

59. (exp Animals/ or exp Animal Experimentation/ or exp Models, Animal/) not Humans/

60. Editorial.pt.

61. 59 or 60

62. 58 not 61

63. 30 and 62

**Embase**1. exp Blood Component Therapy/

2. *Blood Transfusion/

3. (transfus* or pretransfus* or posttransfus* or retransfus* or packed red cells or packed red blood cells or RBC* or PRBC* or packed red blood cells or platelets or fresh plasma or frozen plasma or FFP or platelet concentrate* or blood product* or blood component* or blood management or blood therapy or blood replacement).ti,kw.

4. (pretransfus* or posttransfus* or retransfus* or RBC transfusion* or pRBC transfusion* or red cell transfusion* or red blood cell transfusion* or packed cell transfusion* or blood transfusion* or platelet transfusion* or plasma transfusion* or fresh plasma or frozen plasma or FFP or platelet concentrate* or packed red cell* or packed red blood cell* or ((packed cell* or allogeneic) adj2 transfus*)).ab.

5. ((transfus* or whole blood or component therapy or blood component* or blood product* or blood replacement or allogeneic blood or allogenic blood or red cell* or red blood cell* or thawed plasma or liquid plasma or lyophili?ed plasma or platelets or packed cell* or packed red cell* or packed red blood cell* or RBC* or PRBC*) adj4 (use* or usage* or utili?ation or requir* or replac* or administ* or need* or protocol*)).tw,kw.

6. (((haemostatic or hemostatic) adj1 resuscitation) or haemotherap* or hemotherap*).tw,kw.

7. (total blood volume and transfus*).tw,kw.

8. ((red cell* or red blood cell* or RBC* or PRBC* or blood transfus* or transfus* ratio) adj5 (FFP or fresh plasma or frozen plasma or plasma transfusion*)).tw,kw.

9. or/1-8

10. Hemorrhagic Shock/

11. Hemorrhagic Hypotension/

12. Postpartum Hemorrhage/

13. ((major* or massive* or critical* or severe* or life-threatening or catastrophic* or uncontrolled or postpartum* or trauma*) adj3 (hemorrhag* or haemorrhag* or bleed* or bloodloss* or blood loss*)).tw,kw.

14. ((haemorrhagic or hemorrhagic or hypovolemic or hypovolaemic) adj shock).tw,kw.

15. exsanguinat*.mp.

16. or/10-15

17. Multiple Trauma/

18. exp Traumatic Shock/

19. ((major* or life-threatening or multip* or severe* or serious* or catastrophic* or critical* or catastrophic* or massive* or penetrating) adj3 (trauma* or posttrauma* or postrauma* or injury or injuries or injured or postinjur*)).tw,kw.

20. ((trauma* adj3 coagulopath*) or trauma* resuscitation or polytrauma*).tw,kw.

21. (severe* trauma* or major* trauma* or massive* trauma* or multipl* trauma* or life-threatening trauma* or polytrauma* or severe* injur* or serious* injur* or massive* injur* or critical* injur* or major* injur* or life-threatening injur* or multipl* injur*).ti,kw.

22. or/17-21

23. Aortic Rupture/

24. ((ruptur* or burst*) adj3 aort*).tw,kw.

25. or/23-24

26. 16 or 22 or 25

27. 9 and 26

28. ((massive* or mass or supermassive* or supramassive* or major) adj3 transfus*).tw,kw.

29. ((massive* dos* or massive* volume* or high dos* or high volume* or large dos* or large volume*) adj3 (red cell* or red blood cell* or RBC* or packed red cell* or packed red blood cell* or PRBC* or plasma or FFP or platelet* or transfus*)).tw,kw.

30. ((major bleeding or major haemorrhag* or major hemorrhag* or massive* bleeding or massive* haemorrhag* or massive* hemorrhag*) adj3 (management* or protocol*)).tw,kw.

31. ((trauma* or neurotrauma* or polytrauma* or posttrauma* or substantial* injur* or penetrating injur* or severe* injur* or serious* injur* or massive* injur* or critical* injur* or major* injur* or life-threatening injur* or multipl* injur*) adj6 (transfus* or whole blood or component therapy or blood component* or blood product* or blood replacement or allogeneic blood or allogenic blood or red cell* or red blood cell* or FFP or fresh plasma or frozen plasma or thawed plasma or liquid plasma or platelets or platelet concentrate* or packed cells or RBC* or PRBC*)).tw,kw.

32. or/27-31

33. Meta Analysis/

34. (meta analy* or metaanaly*).ti,kw.

35. ((meta analy* or metaanaly*) and (trials or studies)).ab.

36. Systematic Review/

37. ((systematic* or evidence-based) adj2 (review* or overview*)).tw,kw.

38. (evidence synthes* or cochrane or medline or pubmed or embase or cinahl or cinhal or lilacs or "web of science" or google scholar or google database or science citation index or scopus or search terms or literature search or electronic search* or comprehensive search* or systematic search* or published articles or search strateg* or reference list* or bibliograph* or handsearch* or hand search* or manual* search*).ab.

39. ((electronic* or online) adj (sources or resources or databases)).ab.

40. ((additional adj (papers or articles or sources)) or (relevant adj (journals or articles))).ab.

41. exp "Controlled Clinical Trial (Topic)"/

42. or/33-41

43. Review.pt.

44. (data extraction or selection criteria).ab.

45. 43 and 44

46. 42 or 45

47. Editorial.pt.

48. 46 not 47

49. crossover-procedure/ or double-blind procedure/ or randomized controlled trial/ or single-blind procedure/

50. (random* or factorial* or crossover* or cross over* or cross-over* or placebo* or doubl* blind* or singl* blind* or assign* or allocat* or volunteer*).mp.

51. 48 or 49 or 50

52. (exp animal/ or nonhuman/) not exp human/

53. 51 not 52

54. 32 and 53

**THE COCHRANE LIBRARY**

#1 MeSH descriptor: [Blood Transfusion] explode all trees

#2 (transfus* or pretransfus* or posttransfus* or retransfus* or "packed red cells" or "packed red blood cells" or RBC* or PRBC* or platelets or "fresh plasma" or "frozen plasma" or FFP or "platelet concentrate" or "platelet concentrates" or "blood product" or "blood products" or "blood component" or "blood components" or "blood management" or "blood therapy" or "blood replacement"):ti

#3 (pretransfus* or posttransfus* or retransfus* or "RBC transfusion" or "RBC transfusions" or "pRBC transfusion" or "pRBC transfusions" or "red cell transfusion" or "red cell transfusions" or "red blood cell transfusion" or "red blood cell transfusions" or "packed cell transfusion" or "packed cell transfusions" or "blood transfusion" or "blood transfusions" or "platelet transfusion" or "platelet transfusions" or "plasma transfusion" or "plasma transfusions" or "fresh plasma" or "frozen plasma" or FFP or "platelet concentrate" or "platelet concentrates" or "packed red cell" or "packed red cells" or "packed red blood cell" or "packed red blood cells" or (("packed cell" or "packed cells" or allogeneic) near/2 transfus*)):ab

#4 ((transfus* or "whole blood" or "component therapy" or "blood component" or "blood components" or "blood product" or "blood products" or "blood replacement" or "allogeneic blood" or "allogenic blood" or "red cell" or "red cells" or "red blood cell" or "red blood cells" or "thawed plasma" or "liquid plasma" or "lyophilized plasma" or "lyophilised plasma" or platelet* or "packed cell" or "packed cells" or "packed red cell" or "packed red cells" or "packed red blood cell" or "packed red blood cells" or RBC* or PRBC*) near/4 (use* or usage* or utili?ation or requir* or replac* or administ* or need* or protocol*))

#5 (((haemostatic or hemostatic) near/1 resuscitation) or haemotherap* or hemotherap*)

#6 ("total blood volume" and transfus*)

#7 (("red cell" or "red cells" or "red blood cell" or "red blood cells" or RBC* or PRBC* or "blood transfusion" or "blood transfusions" or "transfusion ratio") near/5 (FFP or "fresh plasma" or "frozen plasma" or "plasma transfusion" or "plasma transfusions"))

#8 #1 or #2 or #3 or #4 or #5 or #6 or #7

#9 MeSH descriptor: [Shock, Hemorrhagic] this term only

#10 exsanguinat*

#11 MeSH descriptor: [Postpartum Hemorrhage] this term only

#12 ((major* or massive* or critical* or severe* or life-threatening or catastrophic* or uncontrolled or postpartum* or trauma*) near/3 (hemorrhag* or haemorrhag* or bleed* or bloodloss* or "blood loss"))

#13 ((haemorrhagic or hemorrhagic or hypovolemic or hypovolaemic) next shock)

#14 #9 or #10 or #11 or #12 or #13

#15 MeSH descriptor: [Multiple Trauma] explode all trees

#16 MeSH descriptor: [Shock, Traumatic] explode all trees

#17 ((major* or life-threatening or multip* or severe* or serious* or catastrophic* or critical* or massive* or penetrating) near/3 (trauma* or posttrauma* or postrauma* or injury or injuries or injured or postinjur*))

#18 ((trauma* near/3 coagulopath*) or (trauma* near/2 resuscitation) or polytrauma*)

#19 ((severe* or major* or massive* or multiple or life-threatening) near/2 (trauma* or injur*)):ti

#20 ((serious* or critical*) near/2 injur*):ti

#21 polytrauma*

#22 #15 or #16 or #17 or #18 or #19 or #20 or #21

#23 MeSH descriptor: [Aortic Rupture] this term only

#24 ((ruptur* or burst*) near/3 aort*)

#25 #23 or #24

#26 #14 or #22 or #25

#27 #8 and #26

#28 ((massive* or mass or supramassive* or supermassive* or major) near/3 transfus*)

#29 (("massive dose" or "massive volume" or "high dose" or "high volume" or "large dose" or "large volume") near/3 ("red cell" or "red cells" or "red blood cell" or "red blood cells" or RBC* or "packed cell" or "packed cells" or PRBC* or plasma or FFP or platelet* or transfus*))

#30 (("major bleeding" or "major bleed" or "major haemorrhage" or "major hemorrhage" or "massive bleeding" or "massive bleed" or "massive haemorrhage" or "massive hemorrhage") near/3 (management* or protocol*))

#31 ((trauma* or neurotrauma* or polytrauma* or posttrauma* or "severe injury" or "severely injured" or "serious injury" or "seriously injured" or "massive injuries" or "massively injured" or "critical injury" or "critically injured" or "major injury" or "major injuries" or "life-threatening injuries" or "life-threatening injury" or "multiple injuries") near/5 (transfus* or "whole blood" or "component therapy" or "blood component" or "blood components" or "blood product" or "blood products" or "blood replacement" or "allogeneic blood" or "allogenic blood" or "red cell" or "red cells" or "red blood cell" or "red blood cells" or FFP or "fresh plasma" or "frozen plasma" or "thawed plasma" or "liquid plasma" or platelets or "platelet concentrate" or "platelet concentrates" or "packed cells" or RBC* or PRBC*))

#32 #27 or #28 or #29 or #30 or #31

**PubMed**

#1 (transfus*[TI] OR pretransfus*[TI] OR posttransfus*[TI] OR retransfus*[TI] OR "packed red cells"[TI] OR "packed red blood cells"[TI] OR RBC*[TI] OR PRBC*[TI] OR platelets[TI] OR "fresh plasma"[TI] OR "frozen plasma"[TI] OR FFP[TI] OR "platelet concentrate"[TI] OR "platelet concentrates"[TI] OR "blood product"[TI] OR "blood products"[TI] OR "blood component"[TI] OR "blood components"[TI] OR "blood management"[TI] OR "blood therapy"[TI] OR "blood replacement"[TI] OR "packed cell"[TI] OR "packed cells"[TI] OR "allogeneic transfusion"[TI] OR "allogeneic transfusions"[TI] OR "allogenic transfusion"[TI] OR "allogenic transfusions"[TI])

#2 ((transfus*[TIAB] OR "whole blood"[TIAB] OR "component therapy"[TIAB] OR "blood component"[TIAB] OR "blood components"[TIAB] OR "blood product"[TIAB] OR "blood products"[TIAB] OR "blood replacement"[TIAB] OR "allogeneic blood"[TIAB] OR "allogenic blood"[TIAB] OR "red cell"[TIAB] OR "red cells"[TIAB] OR "red blood cell"[TIAB] OR "red blood cells"[TIAB] OR "thawed plasma"[TIAB] OR "liquid plasma"[TIAB] OR "lyophilized plasma"[TIAB] OR "lyophilised plasma"[TIAB] OR platelet*[TIAB] OR "packed cell"[TIAB] OR "packed cells"[TIAB] OR "packed red cell"[TIAB] OR "packed red cells"[TIAB] OR "packed red blood cell"[TIAB] OR "packed red blood cells"[TIAB] OR RBC[TIAB] OR RBCs[TIAB] OR PRBC*[TIAB]) AND (use*[TIAB] OR usage*[TIAB] OR utilization[TIAB] OR utilisation[TIAB] OR requir*[TIAB] OR replac*[TIAB] OR administ*[TIAB] OR need*[TIAB] OR protocol*[TIAB]))

#3 (((haemostatic[TIAB] OR hemostatic[TIAB]) AND resuscitation[TIAB]) OR haemotherap*[TIAB] OR hemotherap*[TIAB])

#4 ("total blood volume"[TIAB] AND transfus*[TIAB])

#5 (("red cell"[TIAB] OR "red cells"[TIAB] OR "red blood cell"[TIAB] OR "red blood cells"[TIAB] OR RBC[TIAB] OR RBCs[TIAB] OR PRBC*[TIAB] OR "blood transfusion"[TIAB] OR "blood transfusions"[TIAB] OR "transfusion ratio"[TIAB]) AND (FFP[TIAB] OR "fresh plasma"[TIAB] OR "frozen plasma"[TIAB] OR "plasma transfusion"[TIAB] OR "plasma transfusions"[TIAB]))

#6 #1 OR #2 OR #3 OR #4 OR #5

#7 ("major hemorrhage"[TIAB] OR "massive hemorrhage"[TIAB] OR "severe hemorrhage"[TIAB] OR "life-threatening hemorrhage"[TIAB] OR "catastrophic hemorrhage"[TIAB] OR "uncontrolled hemorrhage"[TIAB] OR "postpartum hemorrhage"[TIAB] OR "traumatic hemorrhage"[TIAB] OR "trauma hemorrhage"[TIAB] OR "major haemorrhage"[TIAB] OR "massive haemorrhage"[TIAB] OR "severe haemorrhage"[TIAB] OR "life-threatening haemorrhage"[TIAB] OR "catastrophic haemorrhage"[TIAB] OR "uncontrolled haemorrhage"[TIAB] OR "postpartum haemorrhage"[TIAB] OR "traumatic haemorrhage"[TIAB] OR "trauma haemorrhage"[TIAB] OR "major bleed"[TIAB] OR "massive bleed"[TIAB] OR "severe bleed"[TIAB] OR "life-threatening bleeding"[TIAB] OR "catastrophic bleed"[TIAB] OR "uncontrolled bleeding"[TIAB] OR "postpartum bleeding"[TIAB] OR "traumatic bleeding"[TIAB] OR "major bloodloss"[TIAB] OR "massive bloodloss"[TIAB] OR "severe bloodloss"[TIAB] OR "life-threatening bloodloss”[TIAB] OR "catastrophic bloodloss"[TIAB] OR "uncontrolled bloodloss"[TIAB])

#8 (exsanguinat*[TIAB] OR "haemorrhagic shock"[TIAB] OR "hemorrhagic shock"[TIAB] OR "hypovolemic shock"[TIAB] OR "hypovolaemic shock"[TIAB] OR "ruptured aorta"[TIAB] OR "aortic rupture"[TIAB] OR "burst aorta"[TIAB])

#9 (trauma*[TIAB] AND (coagulopath*[TIAB] OR resuscitation[TIAB]))

#10 ("severe trauma"[TI] OR "major trauma"[TI] OR "massive trauma"[TI] OR "multiple trauma"[TI] OR "life-threatening trauma"[TI] OR "severe injury"[TI] OR "severely injured"[TI] OR "major injury"[TI] OR "massive injuries"[TI] OR "multiple injuries"[TI] OR "life-threatening injury"[TI] OR "serious injury"[TI] OR "seriously injured"[TI] OR "critical injury"[TI] OR "critically injured"[TI])

#11 #7 OR #8 OR #9 OR #10

#12 #6 AND #11

#13 ("massive transfusion" OR "massive transfusions" OR "massively transfused" OR "major transfusion" OR "major transfusions" OR "mass transfusion" OR "mass transfusions" OR "supermassive transfusion" OR "supermassive transfusions" OR "supramassive transfusion" OR "supramassive transfusions")

#14 (("massive dose"[TI] OR "massive volume"[TI] OR "high dose"[TI] OR "high volume"[TI] OR "large dose"[TI] OR "large volume"[TI]) AND ("red cell"[TI] OR "red cells"[TI] OR "red blood cell"[TI] OR "red blood cells"[TI] OR RBC[TI] OR RBCs[TI] OR "packed cell"[TI] OR "packed cells"[TI] OR PRBC*[TI] OR plasma[TI] OR FFP[TI] OR platelet*[TI] OR transfus*[TI]))

#15 (("major bleeding"[TI] OR "major bleed"[TI] OR "major haemorrhage"[TI] OR "major hemorrhage"[TI] OR "massive bleeding"[TI] OR "massive bleed"[TI] OR "massive haemorrhage"[TI] OR "massive hemorrhage"[TI]) AND (management*[TI] OR protocol*[TI]))

#16 ((trauma*[TI] OR neurotrauma*[TI] OR polytrauma*[TI] OR posttrauma*[TI] OR "severe injury"[TI] OR "severely injured"[TI] OR "serious injury"[TI] OR "seriously injured"[TI] OR "massive injuries"[TI] OR "massively injured"[TI] OR "critical injury"[TI] OR "critically injured"[TI] OR "major injury"[TI] OR "major injuries"[TI] OR "life-threatening injuries"[TI] OR "life-threatening injury"[TI] OR "multiple injuries"[TI]) AND (transfus*[TI] OR "whole blood"[TI] OR "component therapy"[TI] OR "blood component"[TI] OR "blood components"[TI] OR "blood product"[TI] OR "blood products"[TI] OR "blood replacement"[TI] OR "allogeneic blood"[TI] OR "allogenic blood"[TI] OR "red cell"[TI] OR "red cells"[TI] OR "red blood cell"[TI] OR "red blood cells"[TI] OR FFP[TI] OR "fresh plasma"[TI] OR "frozen plasma"[TI] OR "thawed plasma"[TI] OR "liquid plasma"[TI] OR platelets[TI] OR "platelet concentrate"[TI] OR "platelet concentrates"[TI] OR "packed cells"[TI] OR RBC[TI] OR RBCs[TI] OR PRBC*[TI]))

#17 #12 OR #13 OR #14 OR #15 OR #16

#18 (random* OR blind* OR "control group" OR placebo* OR controlled OR groups OR trial* OR "systematic review" OR "meta-analysis" OR metaanalysis OR “evidence synthesis” OR "literature search" OR medline OR pubmed OR cochrane OR embase) AND (publisher[sb] OR inprocess[sb] OR pubmednotmedline[sb])

#19 #17 AND #18

**CINAHL**

S1 (MH "Blood Transfusion+")

S2 TI (transfus* or pretransfus* or posttransfus* or retransfus* or packed red cells or packed red blood cells or RBC* or PRBC* or packed red blood cells or platelets or fresh plasma or frozen plasma or FFP or platelet concentrate* or blood product* or blood component* or blood management or blood therapy or blood replacement)

S3 AB (pretransfus* or posttransfus* or retransfus* or RBC transfusion* or pRBC transfusion* or red cell transfusion* or red blood cell transfusion* or packed cell transfusion* or blood transfusion* or platelet transfusion* or plasma transfusion* or fresh plasma or frozen plasma or FFP or platelet concentrate* or packed red cell* or packed red blood cell* or ((packed cell* or allogeneic) N2 transfus*))

S4 TI ( ((transfus* or whole blood or component therapy or blood component* or blood product* or blood replacement or allogeneic blood or allogenic blood or red cell* or red blood cell* or thawed plasma or liquid plasma or lyophili?ed plasma or platelets or packed cell* or packed red cell* or packed red blood cell* or RBC* or PRBC*) N4 (use* or usage* or utili?ation or requir* or replac* or administ* or need* or protocol*)) ) OR AB ( ((transfus* or whole blood or component therapy or blood component* or blood product* or blood replacement or allogeneic blood or allogenic blood or red cell* or red blood cell* or thawed plasma or liquid plasma or lyophili?ed plasma or platelets or packed cell* or packed red cell* or packed red blood cell* or RBC* or PRBC*) N4 (use* or usage* or utili?ation or requir* or replac* or administ* or need* or protocol*)) )

S5 TI ( (((haemostatic or hemostatic) N1 resuscitation) or haemotherap* or hemotherap*) ) OR AB ( (((haemostatic or hemostatic) N1 resuscitation) or haemotherap* or hemotherap*) )

S6 TI ( (total blood volume and transfus*) ) OR AB ( (total blood volume and transfus*) )

S7 TI ( ((red cell* or red blood cell* or RBC* or PRBC* or blood transfus* or transfus* ratio) N5 (FFP or fresh plasma or frozen plasma or plasma transfusion*)) ) OR AB ( ((red cell* or red blood cell* or RBC* or PRBC* or blood transfus* or transfus* ratio) N5 (FFP or fresh plasma or frozen plasma or plasma transfusion*)) )

S8 S1 OR S2 OR S3 OR S4 OR S5 OR S6 OR S7

S9 (MH "Shock, Hemorrhagic")

S10 TX exsanguinat*

S11 (MH "Postpartum Hemorrhage")

S12 TI ( ((major* or massive* or critical* or severe* or life-threatening or catastrophic* or uncontrolled or postpartum* or trauma*) N3 (hemorrhag* or haemorrhag* or bleed* or bloodloss* or blood loss*)) ) OR AB ( ((major* or massive* or critical* or severe* or life-threatening or catastrophic* or uncontrolled or postpartum* or trauma*) N3 (hemorrhag* or haemorrhag* or bleed* or bloodloss* or blood loss*)) )

S13 TI ( ((haemorrhagic or hemorrhagic or hypovolemic or hypovolaemic) N1 shock) ) OR AB ( ((haemorrhagic or hemorrhagic or hypovolemic or hypovolaemic) N1 shock) )

S14 S9 OR S10 OR S11 OR S12 OR S13

S15 (MH "Multiple Trauma")

S16 (MH "Shock, Traumatic+")

S17 TI ( ((major* or life-threatening or multip* or severe* or serious* or catastrophic* or critical* or massive* or penetrating) N3 (trauma* or posttrauma* or postrauma* or injury or injuries or injured or postinjur*)) ) OR AB ( ((major* or life-threatening or multip* or severe* or serious* or catastrophic* or critical* or massive* or penetrating) N3 (trauma* or posttrauma* or postrauma* or injury or injuries or injured or postinjur*)) )

S18 TI ( ((trauma* N3 coagulopath*) or trauma* resuscitation or polytrauma*) ) OR AB ( ((trauma* N3 coagulopath*) or trauma* resuscitation or polytrauma*) )

S19 TI (severe* trauma* or major* trauma* or massive* trauma* or multipl* trauma* or polytrauma* or life-threatening trauma* or severe* injur* or serious* injur* or massive* injur* or critical* injur* or major* injur* or life-threatening injur* or multipl* injur*)

S20 S15 OR S16 OR S17 OR S18 OR S19

S21 (MH "Aortic Rupture")

S22 TI ( ((ruptur* or burst*) N3 aort*) ) OR AB ( ((ruptur* or burst*) N3 aort*) )

S23 S21 OR S22

S24 S14 OR S20 OR S23

S25 S8 AND S24

S26 TX ((massive* or major) N3 transfus*)

S27 TI ( ((massive* dos* or massive* volume* or high dos* or high volume* or large dos* or large volume*) N3 (red cell* or red blood cell* or RBC* or packed red cell* or packed red blood cell* or PRBC* or plasma or FFP or platelet* or transfus*)) ) OR AB ( ((massive* dos* or massive* volume* or high dos* or high volume* or large dos* or large volume*) N3 (red cell* or red blood cell* or RBC* or packed red cell* or packed red blood cell* or PRBC* or plasma or FFP or platelet* or transfus*)) )

S28 TX ((major bleeding or major haemorrhag* or major hemorrhag* or massive* bleeding or massive* haemorrhag* or massive* hemorrhag*) N3 (management* or protocol*))

S29 TI ( ((trauma* or neurotrauma* or polytrauma* or posttrauma* or substantial* injur* or penetrating injur* or severe* injur* or serious injur* or massive* injur* or critical* injur* or major* injur* or life-threatening injur* or multiple injur*) N6 (transfus* or whole blood or component therapy or blood component* or blood product* or blood replacement or allogeneic blood or allogenic blood or red cell* or red blood cell* or FFP or fresh plasma or frozen plasma or thawed plasma or liquid plasma or platelets or platelet concentrate* or packed cells or RBC* or PRBC*)) ) OR AB ( ((trauma* or neurotrauma* or polytrauma* or posttrauma* or substantial* injur* or penetrating injur* or severe* injur* or serious injur* or massive* injur* or critical* injur* or major* injur* or life-threatening injur* or multiple injur*) N6 (transfus* or whole blood or component therapy or blood component* or blood product* or blood replacement or allogeneic blood or allogenic blood or red cell* or red blood cell* or FFP or fresh plasma or frozen plasma or thawed plasma or liquid plasma or platelets or platelet concentrate* or packed cells or RBC* or PRBC*)) )

S30 S25 OR S26 OR S27 OR S28 OR S29

S31 MH randomized controlled trials

S32 MH double‐blind studies OR MH single‐blind studies OR MH random assignment OR MH pretest‐posttest design OR MH cluster sample

S33 TI (randomised OR randomized* OR controlled study)

S34 AB (random* OR placebo* OR controlled study)

S35 TI (trial)

S36 MH (sample size) AND AB ( (assigned OR allocated OR control) )

S37 MH (placebos)

S38 PT (randomized controlled trial)

S39 AB (control W5 group)

S40 MH (crossover design) OR MH (comparative studies)

S41 AB (cluster W3 RCT)
S42 S31 OR S32 OR S33 OR S34 OR S35 OR S36 OR S37 OR S38 OR S39 OR S40 OR S41

S43 MH animals+

S44 MH (animal studies)

S45 TI (animal model*)

S46 S43 OR S44 OR S45

S47 MH (human)

S48 S46 NOT S47

S49 S42 NOT S48

S50 MH META ANALYSIS

S51 MH SYSTEMATIC REVIEW

S52 TI ( ("meta analysis" OR "meta analyses" OR metaanalys* or meta-analys* OR "systematic review" OR "systematic overview" OR "review of reviews") ) OR AB ( ("meta analysis" OR "meta analyses" OR metaanalys* or meta-analys* OR "review of reviews" OR (systematic* W3 (review* OR overview* OR search*))) )

S53 TI ( ("literature review" OR "literature overview" OR "literature search*") ) OR AB ( ("literature review" OR "literature overview" OR "literature search*") )

S54 AB ( (cochrane OR medline OR pubmed OR embase OR cinahl OR cinhal OR lilacs OR "web of science" OR scopus OR "Google Scholar" OR "science citation index" OR ICTRP OR clinicaltrials*) ) AND AB search*

S55 S50 OR S51 OR S52 OR S53 OR S54

S56 S49 OR S55

**TRANSFUSION EVIDENCE LIBRARY**

Clinical Speciality: Critical Care 0R Obstetrics & Gynaecology

AND

massive OR massively OR major OR postpartum OR exsanguination OR haemorrhagic OR hemorrhagic OR hypovolemic OR hypovolaemic

**ClinicalTrials.gov**Other Terms: "massive transfusion" OR "massively transfused" OR "major transfusion" OR "major hemorrhage" OR "major haemorrhage" OR "massive hemorrhage" OR "massive haemorrhage" OR "critical bleeding" OR "major trauma" OR "multiple trauma" OR "critical injury"

AND Interventional Studies

OR

Other Terms: "hemorrhagic shock" OR "hypovolemic shock" OR polytrauma OR "severe hemorrhage" OR "severe haemorrhage" OR "severe bleeding" OR "aortic rupture" OR "severe postpartum hemorrhage" OR "major postpartum hemorrhage" OR "massive postpartum hemorrhage"

AND Interventional Studies

**WHO ICTRP**

massive transfusion OR massively transfused OR major transfusion OR major hemorrhage OR major haemorrhage OR massive hemorrhage OR massive haemorrhage OR critical bleeding OR major trauma OR multiple trauma OR critical injury

OR

hemorrhagic shock OR hypovolemia OR polytrauma OR severe hemorrhage OR severe haemorrhage OR severe bleeding OR aortic rupture OR severe postpartum hemorrhage OR major postpartum hemorrhage OR massive postpartum hemorrhage
